# Bacterial pathogens and in-hospital mortality in revision surgery for periprosthetic joint infection of the hip and knee: analysis of 346 patients

**DOI:** 10.1186/s40001-023-01138-y

**Published:** 2023-05-19

**Authors:** Filippo Migliorini, Christian David Weber, Andreas Bell, Marcel Betsch, Nicola Maffulli, Vanessa Poth, Ulf Krister Hofmann, Frank Hildebrand, Arne Driessen

**Affiliations:** 1 Department of Orthopaedic, Trauma, and Reconstructive Surgery, RWTH University Hospital, Pauwelsstraße 30, 52074 Aachen, Germany; 2 Department of Orthopaedic and Trauma Surgery, Eifelklinik St. Brigida, 52152 Simmerath, Germany; 3 Department of Orthopaedic and Trauma Surgery, University Hospital of Erlangen, 91054 Erlangen, Germany; 4 Department of Medicine, Surgery and Dentistry, University of Salerno, 84081 Baronissi, Italy; 5 School of Pharmacy and Bioengineering, Faculty of Medicine, Keele University, Stoke On Trent, ST4 7QB UK; 6 Centre for Sports and Exercise Medicine, Barts and the London School of Medicine and Dentistry, Mile End Hospital, Queen Mary University of London, 275 Bancroft Road, London, E1 4DG UK

**Keywords:** Arthroplasty, Pathogens, Mortality, Knee, Hip, Infection

## Abstract

**Introduction:**

The management of periprosthetic joint infections (PJI) of the lower limb is challenging, and evidence-based recommendations are lacking. The present clinical investigation characterized the pathogens diagnosed in patients who underwent revision surgery for  PJI of total hip arthroplasty (THA) and total knee arthroplasty (TKA).

**Methods:**

The present study follows the Strengthening the Reporting of Observational Studies in Epidemiology (STROBE). The institutional databases of the RWTH University Medical Centre of Aachen, Germany, were accessed. The OPS (operation and procedure codes) 5–823 and 5–821 and the ICD (International Statistical Classification of Diseases and Related Health Problems) codes T84.5, T84.7 or T84.8 were used. All patients with PJI of a previous THA and TKA who underwent revision surgery were retrieved and included for analysis.

**Results:**

Data from 346 patients were collected (181 THAs and 165 TKAs). 44% (152 of 346 patients) were women. Overall, the mean age at operation was 67.8 years, and the mean BMI was 29.2 kg/m2. The mean hospitalization length was 23.5 days. 38% (132 of 346) of patients presented a recurrent infection.

**Conclusion:**

PJI remain a frequent cause for revisions after total hip and knee arthroplasty. Preoperative synovial fluid aspiration was positive in 37%, intraoperative microbiology was positive in 85%, and bacteraemia was present in 17% of patients. Septic shock was the major cause of in-hospital mortality. The most common cultured pathogens were Staph. epidermidis, Staph. aureus, Enterococcus faecalis, and Methicillin-resistant Staph aureus (MRSA). An improved understanding of PJI pathogens is important to plan treatment strategies and guide the choice of empirical antibiotic regimens in patients presenting with septic THAs and TKAs.

*Level of Evidence*: Level III, retrospective cohort study.

## Introduction

Arthroplasty aims to restore quality of life in patients with end-stage joint degeneration, fractures, or joint infections [1, 2]. According to the German Arthroplasty Register (EPRD), the number of arthroplasties performed increases yearly [3]. In Germany, 233,424 total hip arthroplasties (THAs) and 187,319 total knee arthroplasties (TKAs) were performed in 2016 [4, 5]. In 2019, the number of THAs and TKAs increased to 243,477 and 193,759, respectively [4, 5]. This indicates an increase of THAs and TKAs of 4% and 3%, respectively. As a direct consequence, the number of revision arthroplasties increased. In Germany, revision arthroplasties of the hip increased by 1% from 2016 to 2019 (35,464 to 35,859) [4, 5]. Revision arthroplasties of the knee increased by 4% from 2016 to 2019 (24,940–25,841) [4, 5]. The most important reasons for revision are implant periprosthetic joint infections (PJI), aseptic loosening, and wearing [3]. Almost one-third of all revisions are performed because of PJIs [3, 6, 7]. According to the time elapsed from implantation to symptom manifestation, PJI can be divided into early (< 4 weeks) and late (> 4 weeks) [8]. Intraoperative direct colonization, hematogenous spread and contamination are the most common modality for infection. A few hours after adhesion to the foreign body surface, bacteria and fungi form a multi-layered structure (immature biofilm), which is then transformed into a stable matrix (mature biofilm) [9]. When such biofilm is mature, small colony variants more resistant to antibiotics and the immune system are formed [10]. Proper treatment may achieve success rates of over 90% [11]. In 2020, Rimke et al. conducted a survey on in-hospital management algorithms for PJI [12]. Early infections were treated in 97.6% of cases following the “DAIR principle” (Debridement, Antibiotics, and Implant Retention). Mobile components of the implant should be replaced. The prerequisites for DAIR are intact soft tissues, stable implant, and the absence of multiple resistant bacteria [9]. For late infections, a one- or two-stage implant replacement is recommended [13]. The degree of maturity of the biofilm plays a decisive role in the recommendation [14]. Whether a one-stage procedure promotes greater outcomes than a two-staged procedure has not yet been fully clarified. The two-stage replacement is the most frequently used procedure in the USA [15]. One-step replacement should only be performed if no multiple resistant bacteria are detected, there are intact soft tissues, and patients who have not undergone multiple revisions [9, 16]. A 12-week course of antibiotic therapy is recommended and should start after intraoperative tissue sampling and debridement [17]. A two-stage replacement either with short (< 3 weeks) or long (> 6 weeks) intervals between implant replacement can be performed depending on pathogens and the quality of soft tissues and bones. If two-stage replacement does not yield a satisfactory result, a three-stage replacement or long-term antibiotic therapy in case of resistant pathogens should be applied. Concomitant antibiotic administration is mandatory. Antibiotic therapy aims to eradicate the infection, avoid microorganism resistance, and prevent biofilm formation [17]. International guidelines and high-level recommendations on the management algorithm for PJI are lacking. Therefore, the present study was conducted to characterise the pathogens identified in patients who underwent revision surgery for PJI of THA or TKA.

## Methods

### Study design

The present study was conducted according to the principles of the Declaration of Helsinki and was approved by the ethics committee of the RWTH Aachen University (project ID EK 121/22). The present study follows the Strengthening the Reporting of Observational Studies in Epidemiology: the STROBE Statement [18]. The present investigation was conducted at the Department of Orthopaedics, Trauma and Reconstructive Surgery, of the University Hospital RWTH Aachen, Germany, and the Department of Orthopaedics of the Eifelklinik St. Brigida of Simmerath, Germany. In August 2022, the clinical databases of the institutions were accessed. For the databases of the German institutions the OPS (operation and procedure codes) 5–823 and 5–821 were used in combination with the ICD (International Statistical Classification of Diseases and Related Health Problems) codes T84.5, T84.7 or T84.8 (Table 1). Patients’ data were included in a Microsoft Excel spreadsheet (version 16.6).

**﻿Table 1:** ICD codes used for the database search

Code	Diagnosis/Procedure
5–823	Revision, replacement and removal of a knee joint arthroplasty
5–821	Revision, replacement and removal of a hip joint arthroplasty
T84.5	Infections and inflammatory reactions caused by a joint arthroplasty
T84.7	Infection and inflammatory reaction from other orthopaedic endoprostheses, implants or transplants
T84.8	Other complications from orthopaedic endoprostheses, implants or transplants

All patients with a PJI of THA or TKA who had undergone THA or TKA revision surgery were retrieved. The inclusion criteria were: arthroplasty of knee or hip; microbiological evidence of pathogen using joint aspiration and/ or intraoperative histologic examination and/ or of blood cultures; the presence of at least one of these signs of inflammation at the joint: heat (calor), pain (dolor), redness (rubor), and swelling (tumor). The exclusion criteria were: any other non-infective ailment in a previously implanted arthroplasty; arthroplasty performed in joints other than knee and hip.

### Data collection

The following data were recorded: gender, age at admission, height, weight and BMI, side, joint and the year of implantation. Data concerning the number and length of hospitalisation and the number of revisions were collected. Information on the type of pathogen was collected. Data on mortality were also retrieved. The perioperative risk was assessed using the American Society of Anaesthesiologists (ASA) score [19].

### Statistical analysis

All analyses were conducted by the main author (FM) using the IBM SPSS Statistics software package, version 25. For descriptive statistics, frequency (amount of events/ number of observations) was used for binary variables. Arithmetic mean and standard deviation were adopted for continuous variables.

## Results

### Patient recruitment

**Fig. 1:**
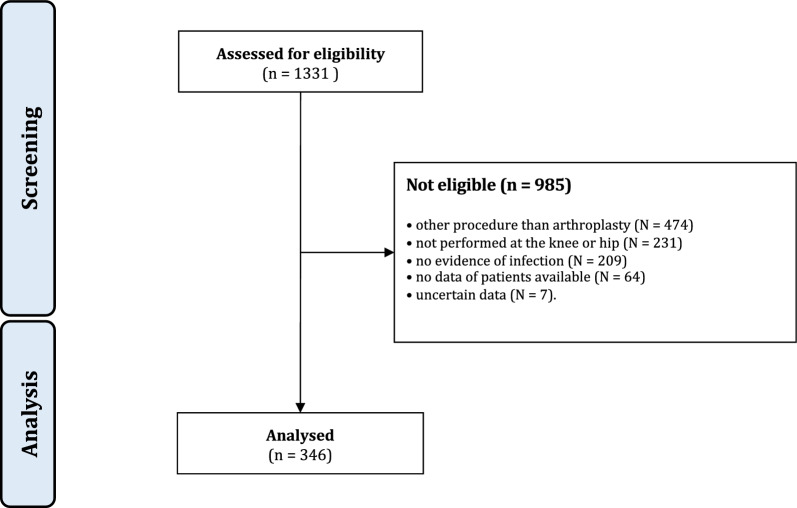
STROBE diagram of the patient recruitment

The database search resulted in 1331 procedures. Of them, 985 procedures were excluded with reason: procedure other than revision arthroplasty (*N* = 474), not performed at the knee or hip (*N* = 231), no evidence of infection (*N* = 209), no data of patients available (*N* = 64), uncertain data (*N* = 7). Finally, 346 patients were considered in the present study (Fig. 1).

### Patient demographic

Data from 346 patients were collected (181 THAs and 165 TKAs). 44% (152 of 346 patients) were women. Overall, the mean age was 67.8 years, the mean BMI 29.2 kg/m^2^. The mean hospitalization length was 23.5 days. 38% (132 of 346) of patients presented a recurrent infection. Demographic information of the patients is shown in Table 2.

**Table 2:** Patient demographics

Endpoint	THA (181)	TKA (165)
Mean age	67.6 ± 23.8	68.1 ± 31.1
Mean BMI	29.3 ± 4.2	29.1 ± 3.9
Women	82 (45%)	71 (43%)
Hospitalisation (*days*)	24 ± 11	23 ± 12
Primary infection	120 (66%)	92 (56%)
Re-infection	61 (34%)	73 (44%)

## Results

In the 346 patients, a maximum of 18 operations were performed per in-hospital stay. In three patients, no operation was performed. On average, 2.6 ± 0.79 revisions per patient were performed. Overall, patients' surgical procedures lasted a mean of 213 ± 183.8 min. The shortest surgical duration was 36 min (one surgical session). The longest surgical duration was 1112 min (13 surgical sessions). Liners were changed in 171 patients; the implants were removed in 155 cases. Amputation was necessary for two patients. Pathogens were detected in 37% (128 of 346) of joint aspirations, 85% (294 of 346) of intraoperative microbiologic examinations, and 17% (59 of 346) of blood cultures. 312 (90%) patients survived, and 34 (10%) patients died during inpatient stay. 31 of these 34 (90%) died from septic shock. One patient died of kidney failure, one of ventricular fibrillation, and one of small cell lung cancer progression (Table 3).

**Table 3:** Frequencies of detected pathogens and patients survivorship

Pathogen (n = 47)	Frequency	Survivorship
Staphylococcus epidermidis	95	89 (94%)
Staphylococcus aureus	77	55 (71%)
Enterococcus faecalis	43	39 (91%)
MRSA	25	20 (80%)
Escherichia coli	22	15 (68%)
Staphylococcus hominis	21	21 (100%)
Streptococcus agalactiae	21	21 (100%)
Staphylococcus haemolyticus	14	14 (100%)
Staphylococcus capitis	13	13 (100%)
Enterococcus faecium	13	8 (62%)
Enterobacter cloacae	11	10 (91%)
Escherichia coli (3 MRGN)	10	8 (10%)
Pseudomonas aeruginosa	9	7 (78%)
Streptococcus dysgalactiae	8	7 (88%)
Staphylococcus warneri	7	7 (100%)
Proteus mirabilis	6	6 (100%)
Streptococcus anginosus	5	5 (100%)
Serratia marcescens (3 MRGN)	5	3 (60%)
Klebsiella pneumoniae	5	4 (80%)
Streptococcus gordonii	4	4 (100%)
Staphylococcus lugudensis	4	4 (100%)
Streptococcus gallolyticus	4	4 (100%)
Finegoldia magna	4	4 (100%)
Citerobacter koseri	3	2 (67%)
Clostridium perfringens	3	3 (100%)
Candida albicans	3	3 (100%)
Corynebacterium tuberculostaticum	3	3 (100%)
Staphylococcus aureus	2	2 (100%)
Peptoniphilus harei	2	2 (100%)
Anaerococcus vaginalis	2	2 (100%)
Staphylococcus caprae	2	2 (100%)
Staphylococcus spp	2	2 (100%)
Probionibacterium spp	2	2 (100%)
Probionibacterium acnes	2	2 (100%)
Corynebacterium amycolatum	2	2 (100%)
Staphylococcus epidermidis (Multiresistent)	1	1 (100%)
Candida parapsiolosis	1	1 (100%)
Spreptococcus pyogenes	1	1 (100%)
Acinteobacter baumanii (4 MRGN)	1	0 (0%)
Granulicatella adiacens	1	1 (100%)
Bacteroides fragilis	1	1 (100%)
bacillus cereus	1	1 (100%)
Enterobacter aerogenes	1	1 (100%)
Acinetobacter johnsonii	1	1 (100%)
Bacillus megaterium	1	1 (100%)
Candida glabrata	1	1 (100%)
Klebsiella oxytoca	1	1 (100%)

*MRSA* Methicillin-resistant Staphylococcus aureus, *MSSA* methicillin-sensitive Staphylococcus aureus, *MRGN* multi-resistant gram negative

## Discussion

PJI are the second most common cause of surgical interventions after arthroplasty of the hip or knee joint, accounting for approximately 15% of all THA and TKA revisions [3]. In 2019, a total of 287 patients died in Germany as a result of a periprosthetic infection [20].

In the present analysis, the spectrum of pathogens in periprosthetic infections was examined in detail. Overall, 175 examined cases showed 66 distinct pathogen combinations with a total of 47 different pathogens. In more than two-thirds of the patients, a single pathogen was detected, and polybacterial infections were less common. This work focused only on the ten most common pathogens with statistical significance on patients’ survival and clinical outcome. An analysis of the remaining pathogens was not reasonable because of their low frequency. Considering *Staphylococcus aureus* and *MRSA* as one group, this was the most frequent microorganism, followed by *Staphylococcus epidermidis* and *Enterococcus faecalis*. To analyze the respective relevance of *Staphylococcus aureus* and methicillin-resistant *Staphylococcus aureus*, these were examined separately. Regarding the prevalence of the detected pathogens, the present study provides similar results compared to those found in the literature [21–25]. However, in the available data, specific pathogens were not further differentiated for either causing TKA or THA infections, which have high mortality rates in revision surgeries [26, 27].

Compared to aseptic revision surgeries, the postoperative mortality risk is markedly increased in septic revisions during the hospitalization period and in the following year [28–30]. Shahi et al. reported higher mortality compared to other major surgical procedures, such as cardiovascular surgery, cholecystectomies, kidney transplants, and carotid surgery. In addition, mortality risk accumulates with each additional revision procedure. Flurin et al. studied 373 cases in which *Staphylococcus epidermidis* was the most frequently detected pathogen. However, in 60% of patients, there was a mixed infection, while an isolated infection accounted for 35% [31].

In the present work, *Staphylococcus epidermidis* was detected in more than half of the patients with mixed infections. Contamination with pathogens of the transient skin flora is possible [32], and its involvement in periprosthetic infections should be clarified. Generally, a distinction between infection and contamination, for example, by molecular genetic testing of the pathogen, is only partially feasible [33]. On the other hand, collecting multiple samples in larger quantities obtained from different sites can be helpful [34]. A negative result for *Staphylococcus epidermidis* in several samples is unlikely for contamination. When interpreting the results, mixed infections from sample contamination with *Staphylococcus epidermidis* must be considered. A significant association between the detection of *Staphylococcus aureus* and a poor clinical outcome could be demonstrated in this work, in accordance with the previous studies. Patients in whom *Staphylococcus aureus* was isolated or detected as part of a mixed infection died significantly more frequently from septic shock than patients with other pathogens. *Escherichia coli* showed a statistically significant association with poor clinical outcomes only when the pathogen was part of a mixed infection. Compared to *Staphylococcus aureus*, which was detected in 39 cases, *Escherichia coli* was found in 12 cases, of which only three were in isolation. Three patients with mixed infections had a lethal septic shock during hospitalization. The limited available data do not allow clarification about the role of Escherichia coli in mixed infections as a determinant of poor clinical outcomes. Significantly higher mortality and the increased prevalence of MRSA compared to MSSA could be demonstrated in the elderly [35]. On the other hand, however, Senneville et al. did not find any difference between MRSA and MSSA regarding clinical outcomes [36]. In addition to MRSA, Fischbacher and Borens described a significantly increased risk of mortality associated with enterococcal infection. Similar results were obtained by Gundtoft et al., where THA infected by enterococci showed higher mortality compared to all other bacteria [37].

Enterococci cause 2–11% of all periprosthetic infections [38], while *Enterococcus faecalis* is more common than *Enterococcus faecium* [38–40]. This distribution is also shown in the present work. In addition, enterococci are detected more frequently in mixed infections and less frequently alone [39–41]. Periprosthetic infections caused by enterococci are difficult to treat and have high therapeutic failure rates from increasing antibiotic resistance [42]. Also, the formation of bacterial biofilms makes them more challenging to treat [43]. Therefore, often several revision surgeries are required [44]. Abdelaziz et al. [45] examined 121 patients after a second revision surgery following PJI. Enterococcal infections were associated with a significantly increased probability of revision after a one-stage procedure [45]. Revision surgery leads to more extended surgery and prolonged hospitalization and consequent poorer outcomes [38, 42]. The present study obtained similar results.

However, the influence of enterococcal infections on mortality was not significant compared to other pathogens. In addition, *Enterococcus faecium* and *Streptococcus agalactiae* were detected frequently in patients with diabetes mellitus, while other pathogens could not be detected significantly more frequently in diabetic patients. However, we acknowledge that our results are limited by the quantitative difference between the individual groups (diabetics = 47 vs. non-diabetics = 128). Considering the high prevalence of diabetics in the present study, diabetes mellitus appears to be a relevant comorbidity for the clinical course [46].

## Conclusion 

Periprosthetic joint infections remain a frequent cause for revisions after total hip and knee arthroplasty. Preoperative synovial fluid aspiration was positive in 37% of patients, intraoperative microbiology was positive in 85% and bacteraemia was present in 17%. Septic shock was the major cause of in-hospital mortality. The most common cultured pathogens were Staph. epidermidis, Staph. aureus, Enterococcus faecalis, and Methicillin-resistant Staph. aureus (MRSA). An improved understanding of PJI pathogens is important to determine treatment strategies and guide the choice of empirical antibiotic regimens in patients presenting with septic THAs and TKAs.

## Data Availability

The data underlying this article are available at reasonable request to the senior author AD (arne.driessen@luisenhospital.de).
